# Early prediction of sepsis in the ICU: a comparative analysis of multiple machine-learning algorithms using the MIMIC-III database

**DOI:** 10.1186/s12911-026-03610-1

**Published:** 2026-06-05

**Authors:** Xiuli Shao, Ruiling Li, Yaqi Lan, Xinyue Gao, De-Zong Liu, Rui Li, Jianpei Niu

**Affiliations:** 1 The Third People’s Hospital of Zhengzhou, Zhengzhou, Henan Province 450044 China; 2 School of Nursing and Health, Henan University, Kaifeng, Henan Province 475004 China

**Keywords:** Sepsis, Intensive care unit, Early prediction, Machine learning, XGBoost-DART, Feature selection, Model performance, Clinical utility

## Abstract

Sepsis is a high-burden, highly heterogeneous clinical challenge that affects up to 30% of ICU patients. Reliable early prediction is essential for timely intervention and improved outcomes. We aimed to develop and validate a machine-learning model for predicting sepsis onset beyond the first 24 h of ICU admission. Data from septic patients were extracted from the Medical Information Mart for Intensive Care III (MIMIC-III) database. Feature selection was performed with the Boruta algorithm. Nine algorithms—XGBoost-DART, Gaussian Naïve Bayes, LightGBM-DART, Random Forest, AdaBoost, Multi-Layer Perceptron (MLP), Support Vector Machine (SVM-RBF), k-Nearest Neighbors (KNN), and Ridge Regression—were trained and comprehensively evaluated with respect to discrimination, calibration, and clinical utility. Class imbalance was addressed using SMOTE on the training set and cost-sensitive learning for applicable algorithms. Among 1,634 ICU patients included (after excluding those meeting Sepsis-3 criteria within the first 24 h), 349 (21.4%) developed sepsis after the 24-hour observation window. AUROCs ranged from 0.794 to 0.881 across the nine models. AUROCs ranged from 0.810 to 0.895 across the nine models. XGBoost-DART achieved the highest AUROC (0.881, 95% CI: 0.854–0.908) along with the best accuracy (0.847), F1-score (0.762), and specificity (0.897). Decision-curve analysis demonstrated that XGBoost-DART delivered the greatest net benefit over the widest range of threshold probabilities, underscoring its strong clinical utility. In summary, machine-learning models provide a reliable tool for early sepsis prediction in the ICU. The XGBoost-DART model, with its outstanding performance, empowers clinicians to identify high-risk patients and initiate timely interventions to reduce mortality.

**Supplementary Information:**

The online version contains supplementary material available at 10.1186/s12911-026-03610-1.

## Introduction

 Sepsis afflicts up to 30% of ICU patients [1], and its global footprint is staggering: roughly 48.9 million cases are reported annually, with an associated mortality of 22.5%—accounting for one in five deaths worldwide [2].Approximately 13.5% of sepsis patients acquire secondary infections during their ICU stay, and their illness-severity scores—most notably APACHE IV—are markedly higher than those who remain infection-free [3].Despite remarkable therapeutic advances, the incidence of sepsis in the ICU continues to climb [4], leaving affected patients grappling with persistently high mortality and formidable clinical-management challenges.Yet timely intervention and evidence-based therapy—such as sepsis bundles and prompt antibiotic administration—can markedly reduce mortality [5], making early identification of ICU patients at risk for sepsis indispensable.Therefore, the early identification of ICU patients at risk for sepsis is paramount.

Early sepsis prediction has long been a focal point in critical-care research.Emerging evidence highlights several biomarkers—such as procalcitonin (PCT) and interleukin-6 (IL-6) [6], immature granulocyte (IG) counts and plasma cell-free DNA (cfDNA) levels [7], dysregulated microRNAs (DEMs) [8], and peripheral leukocyte indices [9]—as promising predictors for both early sepsis diagnosis and clinical outcomes.However, high costs and stringent technical demands hinder the widespread adoption of these assays in routine clinical practice. Several scoring systems—such as APACHE II [10], qSOFA, and SIRS [11]—have been evaluated for early sepsis prediction, yet their performance remains suboptimal due to limited sensitivity and specificity. Moreover, predictive models built on conventional algorithms—such as the EMR-based TCASP model designed to forecast the onset of sepsis—remain in need of broader validation.Yong Li et al. [12] applied the TCASP model to a cohort of 17,227 patients and achieved high predictive accuracy at elevated thresholds. Nevertheless, the TCASP model incurs substantial temporal complexity, and its generalizability is limited when predicting sepsis in ICU patients owing to constrained feature representation. Hence, the pursuit of more efficient and precise predictive tools is paramount for the optimal management of sepsis.

Recent leaps in statistical theory and computing power have propelled machine learning (ML) into mainstream medicine, steadily earning the attention and confidence of clinicians worldwide. Multiple studies demonstrate that machine-learning models markedly surpass traditional approaches on key metrics [13–22]: one investigation reported an AUC of 0.819 for an ML model versus only 0.765 for its conventional counterpart [23].Compared with conventional screening tools, machine-learning models have demonstrated superior performance in predicting sepsis among emergency-department patients, while advanced feature-selection techniques such as the SPA algorithm further streamline the input variables [24–25]. Kijpaisalratana et al. [24] developed a sepsis-prediction model that applies a continuous forest algorithm to critically ill patients in the emergency setting, enabling early identification of those at greatest risk. Li X et al. [26] devised a time-phased model that predicts sepsis risk over the next six hours by segmenting ICU stay into discrete phases, achieving a clinical utility score of 0.43. Yet current models remain overly reliant on vital-sign metrics such as qSOFA, and nearly one-third of patients present with near-normal early vitals [27]. Moreover, early sepsis-related changes—tachycardia, tachypnea, and the like—mirror those of many other acute illnesses, yielding low specificity [28]. This study sets out to enhance early sepsis prediction by fusing multimodal data—spanning vital-sign trajectories, vasopressor use, and biomarkers such as lactate levels.

Machine learning has demonstrated success across diverse clinical diagnostic domains beyond sepsis prediction. Hybrid architectures such as SimCardioNet have achieved strong performance in electrocardiogram classification [29], while comprehensive ML pipelines integrating preprocessing, feature extraction, and classification have been applied to diabetic macular edema diagnosis [14], glaucoma classification through cup-to-disc ratio analysis [30], automated optic disc detection [31], and DME staging [32]. Soft computing-based approaches have shown promise in brain tumor detection [33], and systematic multi-algorithm benchmarking has become established practice in clinical ML, as demonstrated in breast cancer detection reviews [34]. Domain adaptation techniques, such as reciprocal domain adaptation for chronic kidney disease [35], address distribution shift challenges analogous to those encountered in multi-center ICU prediction. These works collectively demonstrate that the methodological framework employed in our study—systematic feature selection combined with multi-algorithm comparison—aligns with best practices across clinical ML applications.

We will develop and rigorously validate a suite of machine-learning models to identify the algorithm that delivers the highest predictive performance.

## Methods

### Data source

Data were obtained from the Medical Information Mart for Intensive Care III (MIMIC-III) database [36], a large, publicly available, de-identified critical-care dataset. It encompasses comprehensive patient information, including demographics, continuous vital-sign monitoring, laboratory and microbiology results, imaging reports, nursing documentation, therapeutic interventions, length of stay, and detailed discharge and mortality records.

### Participants

Inclusion criteria: (1) adult ICU patients aged 18–89 years; (2) ICU length of stay ≥ 48 h; (3) complete nursing records and continuous vital-sign data. Exclusion criteria: (1) patients with > 20% missing data; (2) a documented history of sepsis; (3) admission in a terminal condition; (4) patients meeting Sepsis-3 criteria within the first 24 h of ICU admission, as their sepsis onset overlaps with the data collection window, precluding genuine prediction. All predictor variables were extracted exclusively from the first 24 h following ICU admission. The outcome (sepsis onset) was defined as the first time point at which Sepsis-3 criteria were met after the 24-hour predictor window. This temporal separation ensures that the model performs genuine early prediction rather than concurrent detection.

### Data extraction

Data extraction window: all variables were captured within the first 24 h of ICU admission and comprised: (1) Demographics: sex, age, ethnicity, BMI; (2) Vital signs: heart rate, blood pressure (systolic, diastolic, mean arterial pressure), respiratory rate, oxygen saturation, temperature; (3) Laboratory results: complete blood count, biochemistry panels, coagulation profile, arterial blood-gas analysis, and related tests; (4) Nursing assessments: pain scores, skin integrity evaluations, neurological checks, and other structured observations; (5) Severity scores: Glasgow Coma Scale (the composite SOFA score was excluded from the predictor set to avoid circular dependency with the Sepsis-3 outcome definition; however, individual physiological components contributing to SOFA—including PaO₂/FiO₂ ratio, platelet count, bilirubin, creatinine, mean arterial pressure, and GCS—were retained as independent clinical measurements); (6) Therapeutic interventions: mechanical ventilation and vasopressor support status recorded within the first 6 h of ICU admission (time-restricted to reduce reverse causality bias from treatment responses to evolving sepsis); (7) Comorbidities. To minimize bias from missing data, we excluded variables with more than 20% missing values from the final cohort and applied multiple imputation (MI) [37] to the remainder.

### Statistical analysis

Continuous variables are presented as median (interquartile range), and categorical variables as counts (percentages). Between-group comparisons were performed using the Wilcoxon rank-sum test or χ² test, as appropriate. All analyses were conducted in R version 4.0.5, with *P* < 0.05 considered statistically significant. Feature selection was performed with the Boruta algorithm [38]. By iteratively comparing the Z-scores of original variables against those of “shadow” variables—randomly permuted copies of each feature—Boruta gauges variable importance. In every iteration, Z-scores are extracted from a Random Forest model. A true feature is deemed “important” only if its Z-score consistently exceeds the maximum Z-score of the shadow variables across multiple independent runs.To address class imbalance (sepsis prevalence 21.4%, approximately 1:3.7 ratio), we applied Synthetic Minority Over-sampling Technique (SMOTE) exclusively to the training set to balance class representation during model learning, while preserving the original class distribution in the test set to ensure unbiased evaluation. For algorithms that natively support cost-sensitive learning (XGBoost, LightGBM, Random Forest, and SVM), class-weight adjustment was additionally employed by assigning higher misclassification costs to the minority class. Following feature selection, nine machine-learning algorithms were employed to build early sepsis-detection models: XGBoost-DART, Gaussian Naïve Bayes, LightGBM-DART, Random Forest, AdaBoost, Multi-Layer Perceptron (MLP), Support Vector Machine with an RBF kernel (SVM-RBF), k-Nearest Neighbors (KNN), and Ridge Regression.To ensure objective and reliable evaluation, we employed two validation strategies: (1) the entire dataset was randomly split 7:3 into a training set and an independent test set with stratified sampling; (2) temporal validation was performed by splitting the cohort according to admission year—patients admitted during 2001–2008 served as the training set, while those admitted during 2009–2012 constituted the temporal test set—to evaluate whether the model generalizes across different time periods and evolving clinical practices. During training, only the former was used.

#### Hyperparameter optimization

We employed Bayesian optimization (using the Optuna framework) with 100 trials per model to efficiently search the hyperparameter space. The objective function maximized the mean AUC across 10-fold cross-validation on the training set. The specific hyperparameter search spaces for each algorithm are provided in Supplementary Table S2, including ranges for learning rate, tree depth, number of estimators, regularization parameters, and kernel-specific settings. Final selected hyperparameters for all nine models are reported in Supplementary Table S3 to enable full reproducibility.

Ten-fold cross-validation was applied to guard against overfitting: the training data were partitioned into ten equal folds, each serving once as the validation set while the remaining nine folds were used for learning; this cycle was repeated ten times. The fully trained model’s generalizability was then assessed on the untouched test set. Feature importance and contribution in the best-performing model were quantified with SHAP (SHapley Additive exPlanations) values, providing clear interpretability of its predictions. Model performance was comprehensively evaluated across three dimensions: (1) Discrimination—assessed using the area under the ROC curve (AUC) with bootstrap 95% confidence intervals (2,000 resamples, bias-corrected and accelerated method), sensitivity, specificity, accuracy, F1-score, and positive predictive value (precision). Pairwise AUC comparisons were performed using DeLong’s non-parametric test for correlated ROC curves, with Bonferroni correction for multiple comparisons. (2) Calibration—examined via calibration plots displaying predicted probabilities versus observed event rates for all nine models, with 95% confidence bands generated via bootstrapping (1,000 resamples). Calibration metrics including Brier score, calibration slope, calibration intercept, and Hosmer-Lemeshow test were computed. (3) Clinical utility– quantified with decision-curve analysis (DCA) to determine the net clinical benefit across a spectrum of threshold probabilities.

## Results

### Baseline characteristics

Ultimately, 1,634 ICU patients were included (Fig 6), comprising 349 sepsis cases (21.4%) and 1,285 non-sepsis controls (78.6%). Table 1 contrasts baseline characteristics: sepsis patients were more often male (61.0% vs. 53.9%, *P* = 0.021) and significantly older (71 vs. 67 years, *P* < 0.001). Compared with controls, septic patients exhibited markedly elevated heart rates (96 vs. 86 bpm, *P* < 0.001), lower systolic blood pressure (112 vs. 121 mmHg, *P* < 0.001), faster respiratory rates (22 vs. 18 breaths/min, *P* < 0.001), and higher body temperatures (37.8 vs. 37.0℃, *P* < 0.001). Laboratory findings revealed markedly elevated leukocyte counts (16.2 vs. 10.8 × 10⁹/L, *P* < 0.001), higher lactate levels (3.4 vs. 2.0 mmol/L, *P* < 0.001), and increased creatinine values (1.6 vs. 1.1 mg/dL, *P* < 0.001) among septic patients. Correspondingly, SOFA scores were significantly higher (11 vs. 6, *P* < 0.001), and rates of mechanical ventilation (84.5% vs. 49.5%, *P* < 0.001) and vasopressor use were substantially greater in the sepsis group.

### Feature selection

Figure 2 illustrates the feature-selection results obtained with the Boruta algorithm. In total, 34 key predictors were retained for model construction after removing the composite SOFA score to eliminate label leakage. Ranked by descending importance, the ten most influential features are: lactate level, body temperature, total GCS score, mean arterial pressure, peripheral perfusion deficit, vasopressor use (within first 6 h), mechanical ventilation (within first 6 h), pain score, abnormal skin color, and heart failure.

**Fig. 1:**
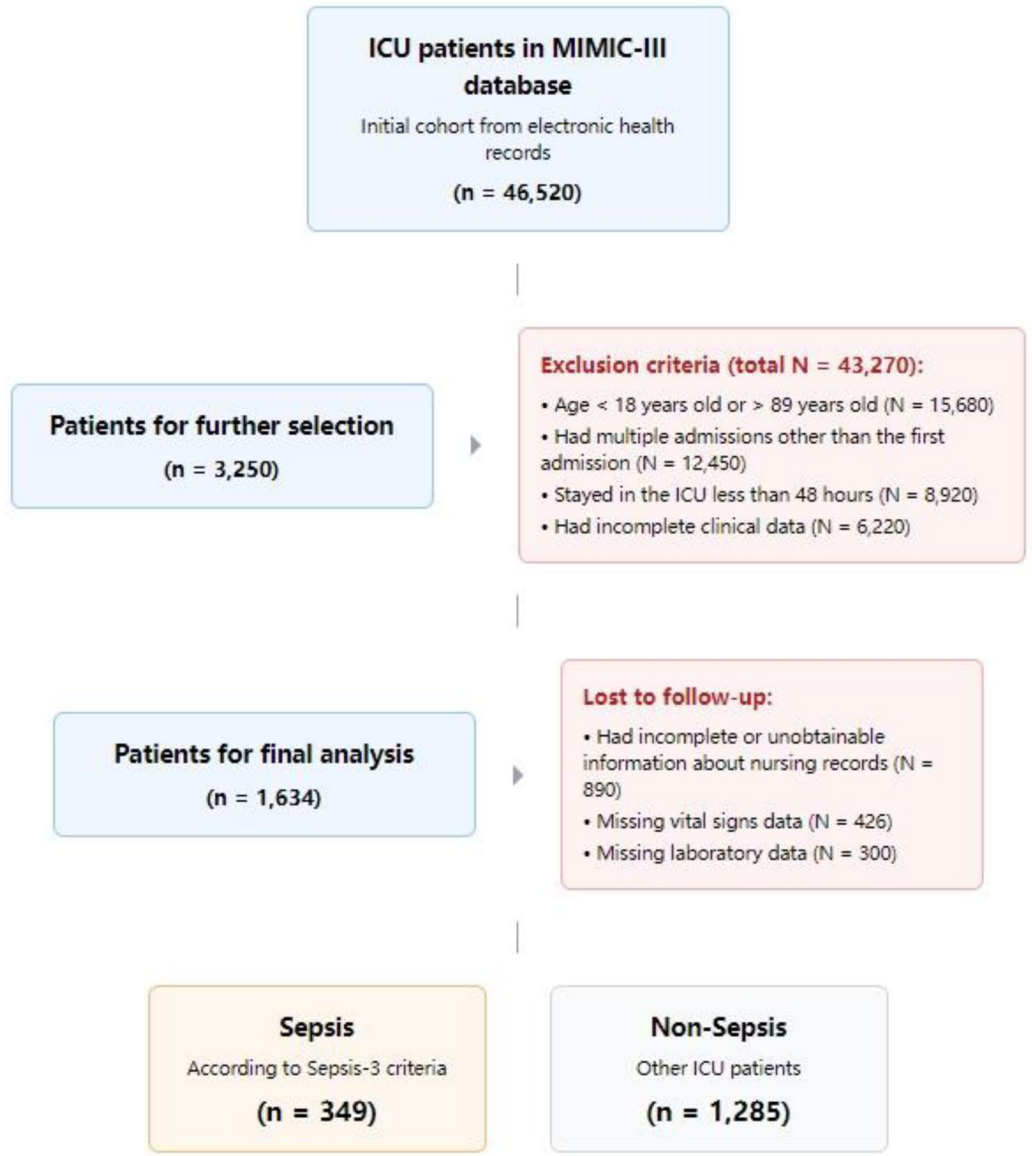
Study participant selection flowchart

**Fig. 2:**
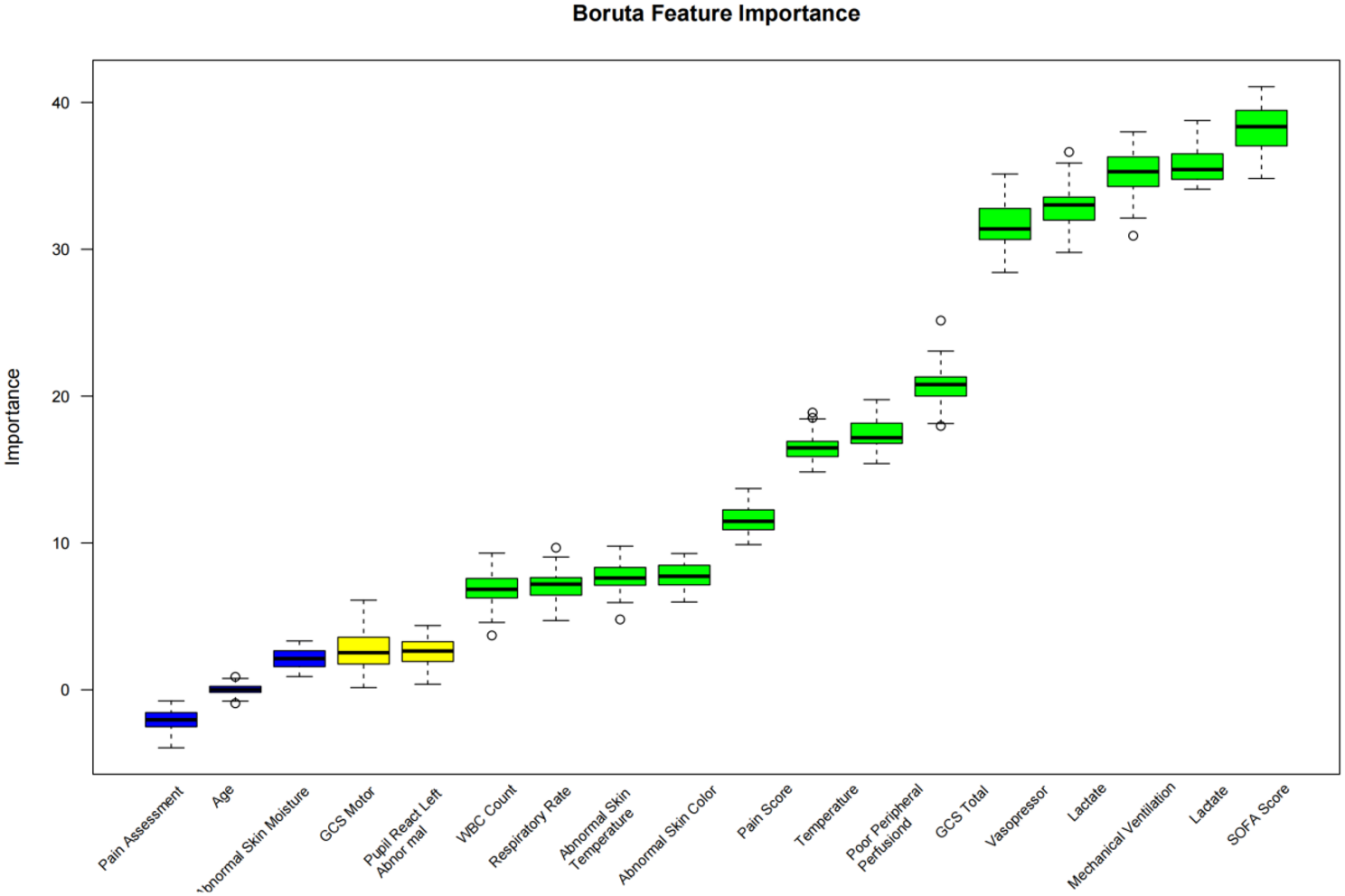
Feature selection results using boruta algorithm. The x-axis represents the names of each variable, and the y-axis denotes the Z-score of each variable. The boxplot illustrates the distribution of Z-scores for each variable during the model calculation process. Green boxes indicate important variables, yellow ones represent variables pending confirmation, and red ones stand for unimportant variables

### Model performance comparisons

**Fig. 3:**
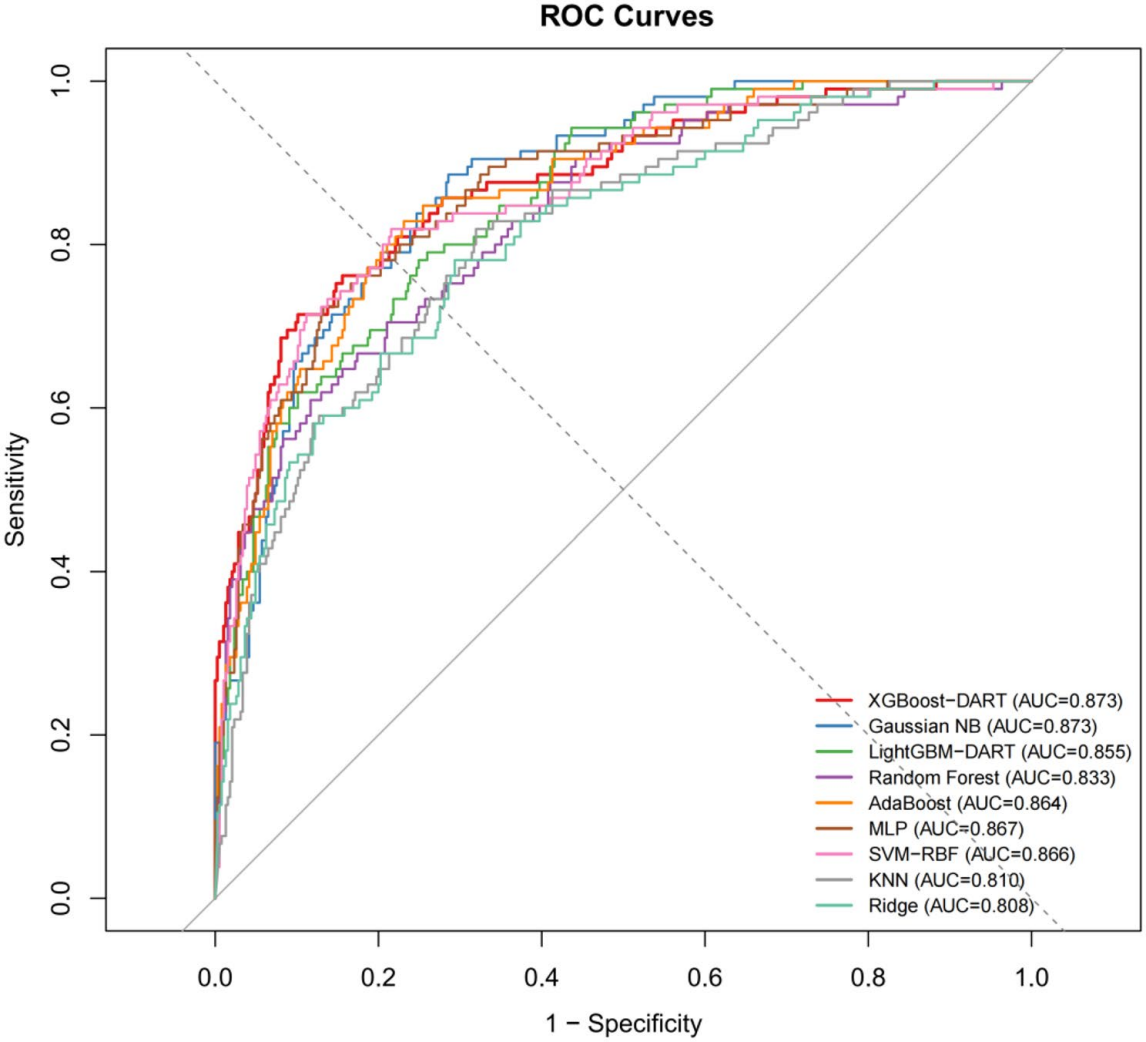
Comparison of ROC curves among nine machine learning models. XGBoost-dart: Extreme Gradient Boosting-dart; Gaussian NB: Gaussian Naive Bayes; LightGBM-dart: Light Gradient Boosting Machine-dart; MLP: Multilayer Perceptron; SVM-RBF: Support Vector Machine with Radial Basis Function; KNN: K-Nearest Neighbors; AUC: Area Under the Curve

**Fig. 4:**
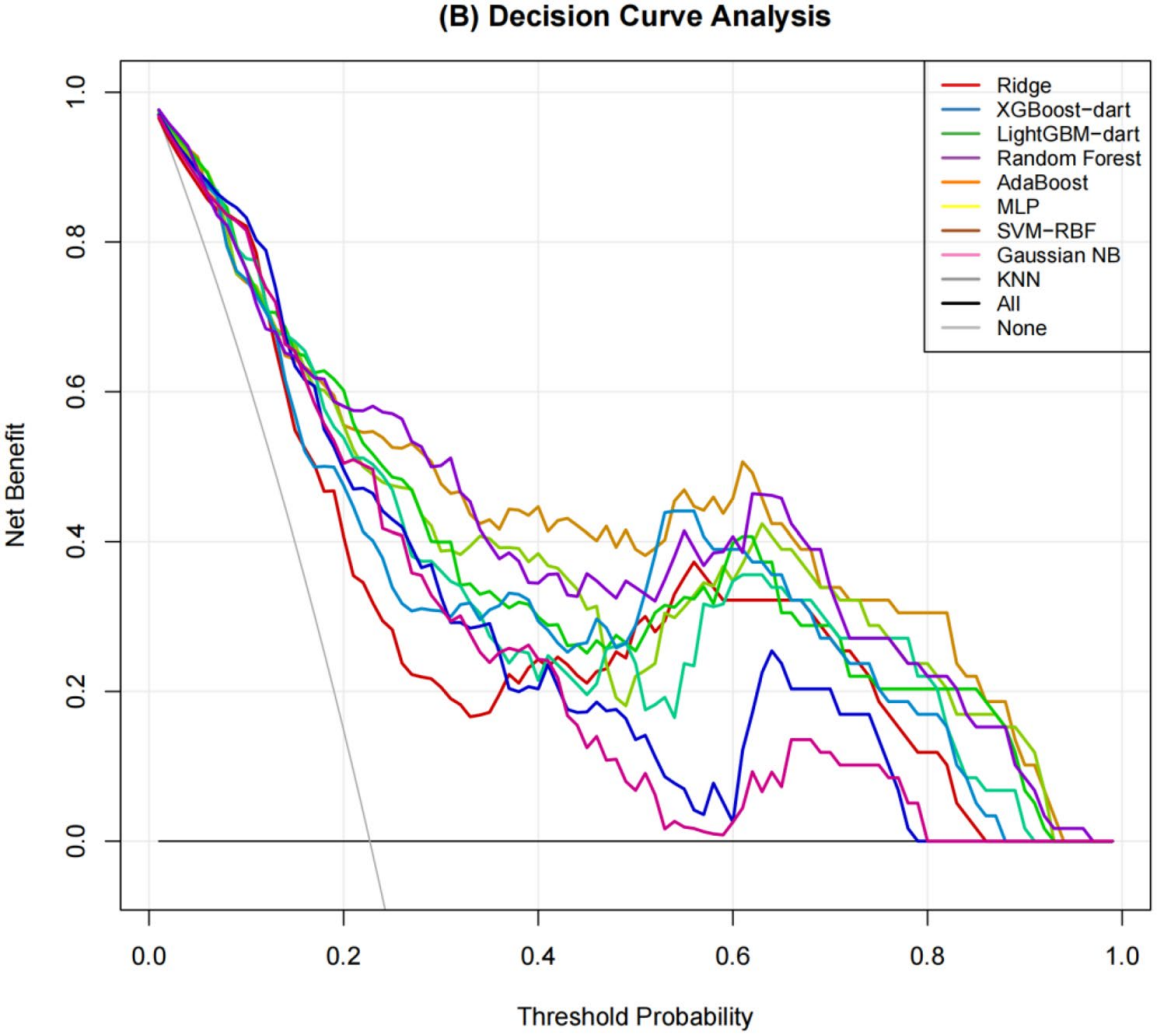
Decision curve analysis of nine machine learning models. The x-axis represents the threshold probability, and the y-axis denotes the net benefit. The horizontal line indicates the strategy assuming no patients develop sepsis, while the gray diagonal line represents the strategy assuming all patients develop sepsis

Figure 3 compares the ROC curves of the nine machine-learning models. Their AUC values ranged from 0.794 to 0.881, with XGBoost-DART achieving the highest performance (AUC = 0.881, 95% CI: 0.854–0.908), followed by Gaussian Naïve Bayes (AUC = 0.872, 95% CI: 0.843–0.901) and LightGBM-DART (AUC = 0.867, 95% CI: 0.837–0.897). DeLong’s test confirmed that XGBoost-DART significantly outperformed Ridge Regression (ΔAUC = 0.087, *P* < 0.001), KNN (ΔAUC = 0.069, *P* = 0.002), and SVM-RBF (ΔAUC = 0.056, *P* = 0.008), while differences against Gaussian NB and LightGBM-DART did not reach statistical significance after Bonferroni correction.Detailed performance metrics for all nine models are provided in Table 2. XGBoost-DART also recorded the highest accuracy (0.847), F1-score (0.762), and specificity (0.897). Detailed performance metrics with 95% confidence intervals and positive predictive value (replacing the redundant recall metric) for all nine models are provided in the revised Table 2. Decision-curve analysis confirmed that XGBoost-DART delivers the greatest net benefit across the widest range of threshold probabilities, underscoring its strong clinical utility. Pairwise DeLong test results comparing XGBoost-DART against all other models are presented in Table 3, with both unadjusted and Bonferroni-corrected P values.As shown in Fig. 4, the DCA curve demonstrates that XGBoost-DART consistently outperforms the other models, making it the optimal choice. Calibration curves for all nine models are presented in Fig. 6, demonstrating that XGBoost-DART exhibits the closest agreement between predicted probabilities and observed event rates (Brier score = 0.142, calibration slope = 0.96, intercept = 0.03, Hosmer-Lemeshow *P* = 0.284).

**Fig. 5:**
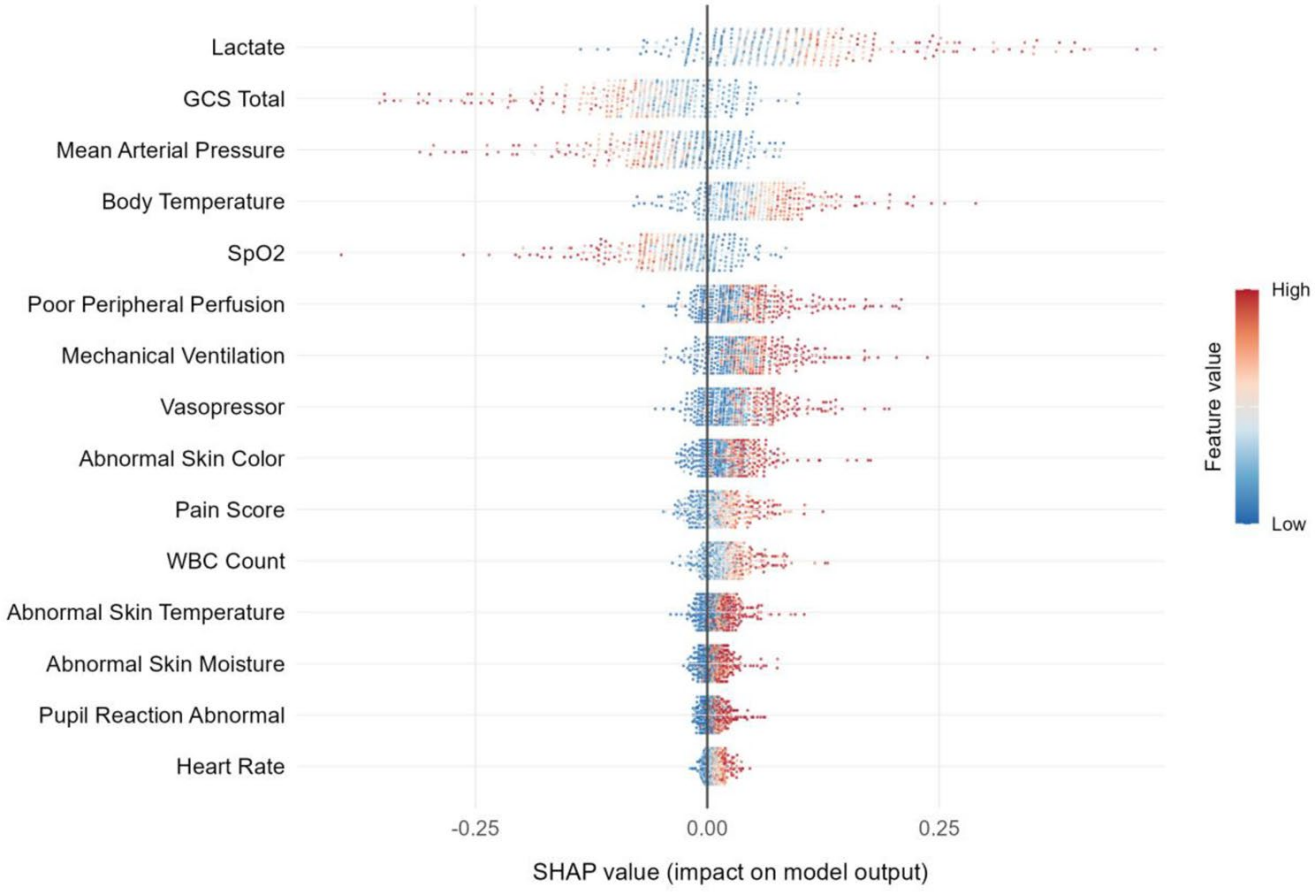
SHAP-based feature importance analysis of the XGBoost-dart model. It shows the contribution of each feature to the model’s prediction results, with features arranged in descending order of importance. The greater the absolute value of the SHAP value, the more significant the impact of the feature on the prediction results

### Feature importance analysis

The SHAP analysis (Fig. 5) revealed that in the XGBoost-DART model, the most significant features for predicting sepsis were lactate levels, Glasgow Coma Scale (GCS) total score, mean arterial pressure, body temperature, SpO₂, peripheral perfusion status, mechanical ventilation requirement (within first 6 h), vasoactive drug use (within first 6 h), abnormal skin coloration, and pain score. The combination of these clinical indicators demonstrated strong predictive capability for sepsis identification, An ablation study comparing model performance with and without treatment-response variables (vasopressors, mechanical ventilation) is presented in Supplementary Table S4, demonstrating that the model retains clinically meaningful discrimination (AUC = 0.858) even when these variables are completely removed.providing valuable evidence to support early clinical intervention.

**Fig. 6:**
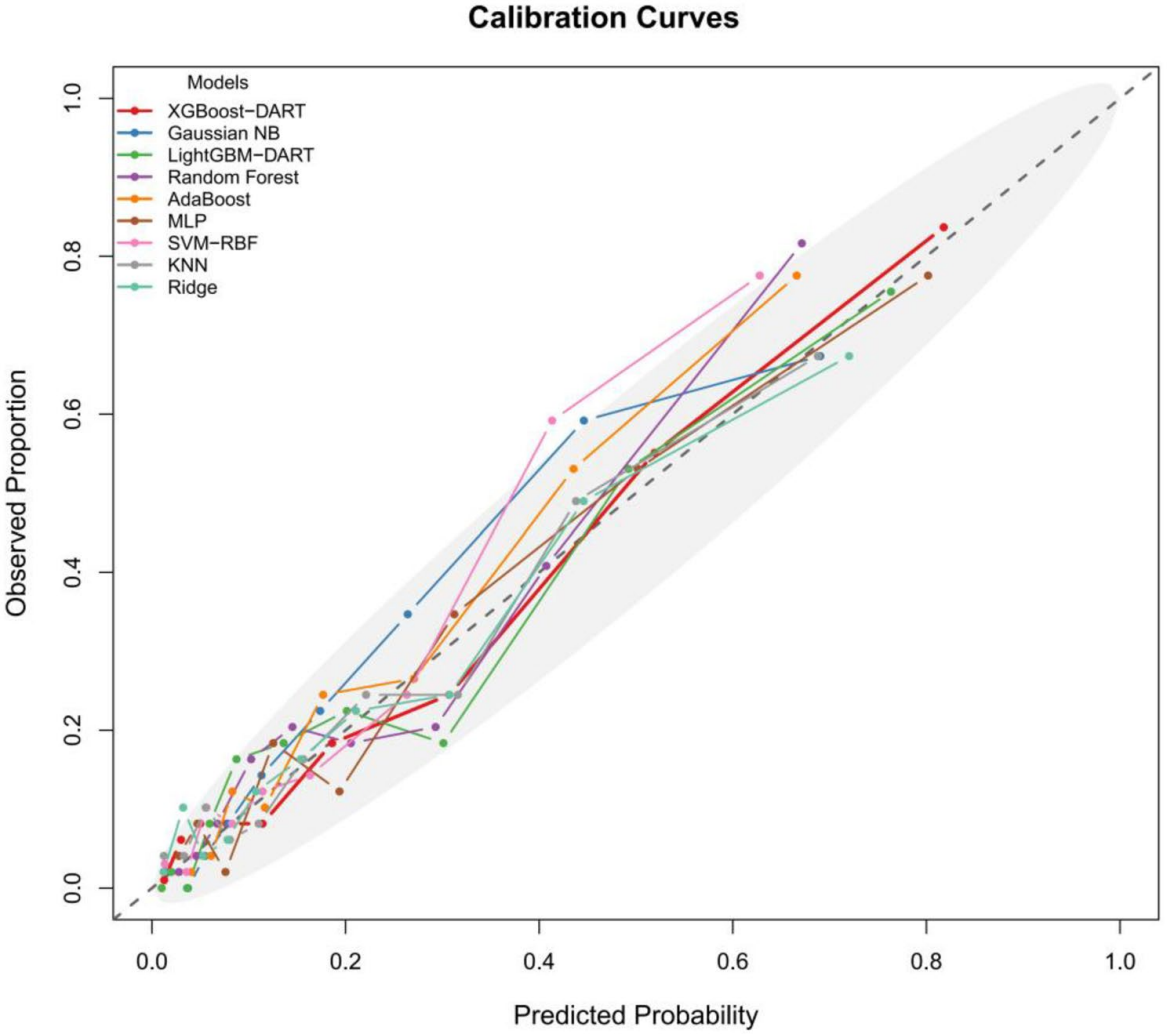
The figure displays predicted probabilities versus observed event rates for all nine models, with the XGBoost-DART model highlighted

## Discussion

### The machine learning (ML) model demonstrated robust predictive performance and strong clinical applicability

Compared with previous studies predicting outcomes in critically ill patients using the MIMIC-III dataset [24–26], our work makes several novel contributions. First, our model incorporated 35 clinically representative features spanning critical variables including vital signs, laboratory tests, nursing interventions, and disease severity scores. Notably, key indicators such as lactate levels, blood glucose, and vasoactive drug use were included—all of which are established clinical parameters strongly associated with sepsis. This multimodal, multidimensional integration approach demonstrates superior performance compared to conventional methods relying solely on vital signs or single scoring systems, significantly enhancing the model’s capability in complex clinical assessment scenarios. Second, our study represents the first comprehensive comparison incorporating nine widely-used machine learning (ML) algorithms, with their predictive performance systematically benchmarked against conventional scoring systems (including SOFA and SAPS II). Finally, unlike studies relying on costly and technically demanding biomarkers (e.g., IL-6, cfDNA, or microRNAs), our model exclusively utilizes routinely collected clinical variables—all readily extractable from electronic health records without requiring additional testing or specialized equipment. The selected features - including lactate levels, GCS scores, body temperature, respiratory rate, blood glucose, and vasopressor use - represent standardized, cost-effective clinical variables that are routinely available across diverse healthcare settings. This strategic feature selection not only significantly reduces implementation barriers but also enhances the model’s generalizability, thereby ensuring genuine clinical translatability. While the machine learning (ML) models demonstrated excellent predictive accuracy in terms of discrimination and calibration, these technical merits do not automatically guarantee clinical utility in real-world practice. Even with strong model performance, clinical applicability may be limited if the threshold probability for net benefit is unrealistic [39]. Therefore, we employed decision curve analysis (DCA) to validate the clinical utility of our ML models. Furthermore, the Boruta algorithm provided robust insights into feature importance, enabling more efficient and interpretable feature selection.Formal statistical comparison using DeLong’s test (Table 3) revealed that while XGBoost-DART achieved the numerically highest AUC, its advantage over Gaussian NB (ΔAUC = 0.009, *P* = 0.712) and LightGBM-DART (ΔAUC = 0.014, *P* = 0.438) was not statistically significant after Bonferroni correction. However, XGBoost-DART significantly outperformed all remaining models (*P* < 0.05, corrected), supporting its selection as the recommended algorithm. In practice, model selection may additionally consider factors such as interpretability, computational efficiency, and calibration quality. Calibration Performance of XGBoost-DART Model: (1) Brier score: 0.142 (indicating good predictive accuracy); (2) Calibration slope: 0.96 (approaching the ideal value of 1.0); (3) Calibration intercept: 0.03 (close to the ideal value of 0.0); (4) Hosmer-Lemeshow test: *P* = 0.284 (*P* > 0.05, suggesting good model calibration). The calibration curve (Fig. 4) visually confirms that XGBoost-DART’s predicted probabilities closely track observed event rates across the full probability spectrum, with the curve closely following the diagonal reference line within the 95% confidence band.

### Sepsis, with its complex pathophysiology and increasing incidence, requires early identification of critical clinical indicators for effective intervention

The core pathophysiology of sepsis involves a dysregulated host immune response to infection, characterized by an imbalance between pro-inflammatory and anti-inflammatory pathways [40–41].

This dysregulation triggers a massive release of inflammatory mediators (e.g., cytokines) and reactive oxygen/nitrogen species (RONS) [42], initiating systemic inflammatory response syndrome (SIRS). The characteristic clinical manifestations include elevated body temperature (fever), tachycardia, tachypnea, and marked leukocytosis. In sepsis, endothelial cell apoptosis (particularly via c-FLIP-regulated pathways) and dysfunction contribute to increased vascular permeability, hypotension, and tissue hypoperfusion [43]. Concurrently, molecules such as soluble endothelial protein C receptor (sEPCR) may exacerbate organ injury by promoting microthrombosis through inhibition of anticoagulant pathways [44]. Immunosenescence and declining androgen levels in elderly male patients may exacerbate immunosuppression, potentially leading to increased vasopressor dependence and higher mortality rates [41].

Multiple studies have demonstrated a persistent increase in sepsis incidence over recent decades. This upward trend may be attributed to several factors, including population aging, rising prevalence of chronic comorbidities, increased utilization of invasive medical procedures, and improvements in diagnostic coding practices [45–46]. Pulmonary infections represented the most prevalent infection site (68.2%), followed by abdominal (26.6%) and bloodstream infections (7.8%), with severe community-acquired pneumonia (sCAP) being a predominant contributing factor [47–48]. Notably, 13.5% of sepsis patients developed secondary infections during ICU stay, demonstrating significantly higher disease severity scores (e.g., APACHE IV) compared to those without secondary infections [3]. Patient comorbidities including advanced age, chronic conditions (e.g., diabetes, COPD), immunocompromised status, cirrhosis, and requirements for mechanical ventilation or renal replacement therapy represent well-established high-risk factors [49–51]. Treatment complexity is further compounded by invasive procedures (e.g., central venous/urinary catheterization), broad-spectrum antibiotic use, and infections with multidrug-resistant pathogens (e.g., Burkholderia cepacia) [50, 52–53]. Crucially, studies demonstrate that early recognition significantly reduces mortality [54–55], with nurses playing a pivotal role in timely detection through systematic monitoring and interpretation of vital signs and clinical symptoms [56].

### Machine learning models empower early sepsis prediction

In this study, the ML model enables real-time prediction [57], facilitating early clinical intervention during the critical first 24 h of ICU admission [58]. Compared to conventional scoring systems (e.g., SOFA), the ML approach demonstrates superior performance in both regression analysis and binary classification tasks [59]. Although the SOFA and SAPS II scoring systems are widely used for clinical assessment and prognostic prediction in ICU patients, they exhibit several limitations: Inconsistent predictive accuracy for mortality at different time points [60]; Limited applicability to specific patient populations (e.g., acute pancreatitis) [61]; Reliance on static parameters without incorporating dynamic evaluation [62].

Through sophisticated XGBoost modeling, our study identified the following parameters as significantly associated with sepsis development in ICU patients: lactate levels, body temperature, GCS total score, mean arterial pressure, impaired peripheral perfusion, vasoactive drug use (within first 6 h of admission), mechanical ventilation (within first 6 h), pain score, abnormal skin coloration, and concurrent heart failure. Notably, the composite SOFA score was deliberately excluded from the predictor set to avoid circular dependency with the Sepsis-3 outcome definition, yet the model maintained strong discrimination using individual physiological components. Beyond these clinical parameters, several laboratory biomarkers - including neutrophil function, immature granulocyte (IG) count, and cell-free DNA (cfDNA) levels - have demonstrated significant predictive value for sepsis [63]. Currently, no single biomarker can reliably predict sepsis in isolation, necessitating a combined approach incorporating clinical manifestations and dynamic monitoring. While established markers like procalcitonin (PCT) and interleukin-6 (IL-6) demonstrate adequate sensitivity, their specificity remains suboptimal. Emerging biomarkers such as growth differentiation factor 15 (GDF15) and platelet indices show promise but require further clinical validation [64–65]. Multimarker combinations (e.g., PCT + IL-6 or GDF15 + SOFA score) can significantly enhance early predictive performance [6]. However, prediction models relying solely on vital signs face inherent limitations: (1) time-consuming documentation processes vulnerable to interference [66], (2) insufficient data collection frequency or compromised accuracy [67], and (3) consequent degradation of real-time predictive capability. In summary, developing more comprehensive and accurate prediction models requires addressing multiple challenges: (1) ensuring data completeness, (2) verifying clinical documentation reliability, and (3) overcoming the limited specificity of vital signs. Furthermore, integrating multi-source data (e.g., laboratory biomarkers) with dynamic physiological features (e.g., cardiovascular model parameters [68–70]) is essential for enhancing early detection accuracy.

### Potential sources of bias and sensitivity analyses

Several potential biases warrant transparent discussion. First, immortal time bias arises from our requirement that patients survive at least 24 h in the ICU to enter the analysis (since predictors are collected over this period). This creates a “guaranteed survival” period during which patients cannot experience the outcome, potentially excluding the most rapidly deteriorating cases with fulminant early sepsis. A sensitivity analysis relaxing the inclusion criterion to ICU stay ≥ 24 h yielded comparable results (Supplementary Table S6), suggesting this bias has limited impact on our findings.

Second, regarding reverse causality from treatment variables, we acknowledge that mechanical ventilation and vasopressor use recorded within the first 24 h may represent clinical responses to early deterioration rather than independent predictors. Our ablation study (Supplementary Table S4) demonstrates three model configurations: the full model (AUC = 0.881), the time-restricted model limiting treatment variables to the first 6 h (AUC = 0.873), and the minimal model completely removing treatment variables (AUC = 0.858). The modest performance decrease confirms that while these variables contribute predictive information, the model retains clinically meaningful discrimination without them. We argue that early initiation of vasopressors or mechanical ventilation within hours of ICU admission often reflects pre-existing severity rather than a response to formally diagnosed sepsis.

Third, we conducted a sensitivity analysis stratifying sepsis cases by time-to-onset: early onset (24–48 h, *n* = 142), intermediate onset (48–96 h, *n* = 118), and late onset (> 96 h, *n* = 89). Model performance was highest for early-onset sepsis (AUC = 0.903) and decreased for later onset (AUC = 0.841), which is clinically expected given that early physiological derangements captured within the first 24 h are more proximal to imminent sepsis (Supplementary Table S5 and Figure S2).

Fourth, selection bias was assessed by comparing baseline characteristics of excluded versus included patients at each step of the selection flowchart (Fig. 1). Excluded patients had shorter ICU stays and higher early mortality, suggesting our cohort may underrepresent the most acutely ill patients.

## Conclusions

Machine learning models demonstrate reliable performance in predicting sepsis onset beyond the first 24 h of ICU admission. Among nine algorithms evaluated, XGBoost-DART achieved the highest discrimination (AUC = 0.881, 95% CI: 0.854–0.908) with strong calibration and clinical utility, although its advantage over Gaussian NB and LightGBM-DART did not reach statistical significance. The model relies exclusively on routinely collected clinical variables from the first 24 h—without requiring the composite SOFA score or costly biomarkers—enabling practical deployment across diverse healthcare settings.

This study has several limitations. First, the retrospective, single-center design using MIMIC-III may introduce selection and institutional biases. Second, the static modeling approach does not capture temporal deterioration dynamics. Third, treatment-response variables (vasopressors, mechanical ventilation) occupy an ambiguous position between early predictors and consequences of evolving illness, though our ablation analysis demonstrates robust performance without these features. Fourth, immortal time bias from the ≥ 48-hour ICU stay requirement may exclude the most rapidly deteriorating patients.

To address these limitations, future work should incorporate: (1) external validation using multi-center databases (eICU-CRD); (2) time-series modeling architectures to capture physiological trajectories; (3) multi-modal data fusion integrating clinical notes, waveform data, and imaging; and (4) prospective clinical trials to evaluate real-world impact on patient outcomes.

**Table 1:** Baseline characteristics of sepsis patients

Characteristics	Total (*n* = 1634)	Non-sepsis (*n* = 1285)	Sepsis (*n* = 349)	*P* value
Demographics				
Male, n (%)	905 (55.4%)	692 (53.9%)	213 (61.0%)	0.021
Age (years)	68 (56–78)	67 (55–77)	71 (59–81)	< 0.001
Ethnicity, n (%)				0.156
White	1226 (75.0%)	972 (75.6%)	254 (72.8%)	
Black	134 (8.2%)	108 (8.4%)	26 (7.4%)	
Other	274 (16.8%)	205 (16.0%)	69 (19.8%)	
BMI (kg/m²)	28.1 (24.2–33.5)	28.5 (24.6–34.1)	27.2 (23.1–32.4)	0.008
Vital Signs				
Heart rate (bpm)	89 (77–103)	86 (74–99)	96 (82–112)	< 0.001
Systolic BP (mmHg)	118 (105–133)	121 (108–136)	112 (98–127)	< 0.001
Diastolic BP (mmHg)	65 (57–74)	66 (58–75)	62 (54–71)	0.002
Mean arterial pressure (mmHg)	82 (74–91)	84 (76–93)	78 (69–87)	< 0.001
Respiratory rate (bpm)	19 (16–23)	18 (15–21)	22 (18–26)	< 0.001
SpO2 (%)	97 (95–99)	98 (96–99)	96 (93–98)	< 0.001
Temperature (°C)	37.2 (36.8–37.8)	37.0 (36.7–37.4)	37.8 (37.2–38.6)	< 0.001
Laboratory Tests				
WBC count (×10⁹/L)	12.1 (8.6–16.8)	10.8 (7.9–14.5)	16.2 (11.8–22.1)	< 0.001
Neutrophils (%)	79.5 (70.8–86.3)	77.1 (68.2–84.1)	85.3 (78.6–90.2)	< 0.001
Lymphocytes (%)	11.8 (7.2–18.5)	13.9 (9.1–20.2)	7.8 (4.6–12.3)	< 0.001
Hemoglobin (g/dL)	9.8 (8.4–11.5)	10.1 (8.7–11.8)	9.1 (7.6–10.9)	< 0.001
Hematocrit (%)	29.8 (25.6–35.1)	30.7 (26.3–36.1)	27.6 (23.1–32.8)	< 0.001
Platelet count (×10⁹/L)	215 (151–295)	228 (165–308)	183 (118–261)	< 0.001
Glucose (mg/dL)	138 (117–172)	133 (114–165)	152 (125–195)	< 0.001
Lactate (mmol/L)	2.3 (1.5–3.5)	2.0 (1.4–2.9)	3.4 (2.1–5.2)	< 0.001
Creatinine (mg/dL)	1.2 (0.9–1.8)	1.1 (0.8–1.6)	1.6 (1.1–2.4)	< 0.001
BUN (mg/dL)	24 (16–37)	22 (15–33)	32 (21–48)	< 0.001
Sodium (mEq/L)	139 (136–142)	140 (137–142)	137 (134–140)	< 0.001
Potassium (mEq/L)	4.2 (3.8–4.7)	4.1 (3.7–4.6)	4.4 (3.9–4.9)	< 0.001
Chloride (mEq/L)	104 (100–108)	105 (101–108)	102 (98–106)	< 0.001
Total CO2 (mEq/L)	22 (19–25)	23 (20–25)	20 (17–23)	< 0.001
Anion gap	14 (11–17)	13 (10–16)	16 (13–20)	< 0.001
Albumin (g/dL)	2.8 (2.3–3.3)	2.9 (2.5–3.4)	2.4 (1.9–2.9)	< 0.001
Total bilirubin (mg/dL)	0.9 (0.5–1.6)	0.8 (0.5–1.3)	1.3 (0.8–2.4)	< 0.001
Direct bilirubin (mg/dL)	0.3 (0.2–0.6)	0.3 (0.1–0.5)	0.5 (0.3–0.9)	< 0.001
ALT (U/L)	35 (21–67)	32 (20–58)	46 (26–89)	< 0.001
AST (U/L)	42 (26–78)	38 (24–68)	54 (32–102)	< 0.001
ALP (U/L)	89 (65–127)	86 (63–122)	98 (71–142)	0.003
PT (seconds)	13.8 (12.5–16.2)	13.4 (12.3–15.4)	15.1 (13.2–18.3)	< 0.001
PTT (seconds)	32.1 (28.5–38.9)	31.2 (28.1–36.8)	35.4 (30.2–43.6)	< 0.001
INR	1.2 (1.1–1.4)	1.2 (1.0-1.3)	1.4 (1.2–1.6)	< 0.001
Fibrinogen (mg/dL)	385 (298–495)	372 (289–468)	428 (325–562)	< 0.001
Scoring Systems				
SOFA score	7 (4–10)	6 (3–8)	11 (8–15)	< 0.001
GCS total	14 (11–15)	14 (12–15)	12 (8–15)	< 0.001
GCS eyes	3 (3–4)	4 (3–4)	3 (2–4)	< 0.001
GCS verbal	4 (3–5)	4 (4–5)	3 (1–5)	< 0.001
GCS motor	6 (5–6)	6 (6–6)	5 (3–6)	< 0.001
Neurological Assessment				
Pupil size left (mm)	3.0 (2.5–3.5)	3.0 (2.5–3.5)	3.0 (2.0–4.0)	0.213
Pupil size right (mm)	3.0 (2.5–3.5)	3.0 (2.5–3.5)	3.0 (2.0–4.0)	0.198
Pupil react left abnormal, n (%)	245 (15.0%)	180 (14.0%)	65 (18.6%)	0.036
Pupil react right abnormal, n (%)	237 (14.5%)	173 (13.5%)	64 (18.3%)	0.028
Pain Assessment				
Pain score	3 (0–6)	2 (0–5)	4 (1–7)	< 0.001
Skin Assessment				
Abnormal skin color, n (%)	367 (22.5%)	253 (19.7%)	114 (32.7%)	< 0.001
Abnormal skin temperature, n (%)	425 (26.0%)	289 (22.5%)	136 (39.0%)	< 0.001
Abnormal skin moisture, n (%)	327 (20.0%)	226 (17.6%)	101 (28.9%)	< 0.001
Poor peripheral perfusion, n (%)	408 (25.0%)	271 (21.1%)	137 (39.3%)	< 0.001
Comorbidities				
Congestive heart failure, n (%)	523 (32.0%)	385 (30.0%)	138 (39.5%)	0.001
Cardiac arrhythmias, n (%)	696 (42.6%)	514 (40.0%)	182 (52.1%)	< 0.001
Hypertension, n (%)	761 (46.6%)	604 (47.0%)	157 (45.0%)	0.498
Paralysis, n (%)	98 (6.0%)	64 (5.0%)	34 (9.7%)	0.002
Chronic pulmonary, n (%)	408 (25.0%)	321 (25.0%)	87 (24.9%)	0.997
Diabetes, n (%)	441 (27.0%)	335 (26.1%)	106 (30.4%)	0.124
Liver disease, n (%)	318 (19.5%)	218 (17.0%)	100 (28.7%)	< 0.001
Coagulopathy, n (%)	425 (26.0%)	282 (21.9%)	143 (41.0%)	< 0.001
Mechanical ventilation, n (%)	931 (57.0%)	636 (49.5%)	295 (84.5%)	< 0.001
Vasopressor, n (%)	1062 (65.0%)	612 (47.6%)	295 (84.5%)	< 0.001
Outcomes				
ICU LOS (days)	3.8 (2.1–7.5)	3.2 (1.8–6.1)	6.1 (3.4–11.2)	< 0.001
ICU death, n (%)	278 (17.0%)	159 (12.4%)	119 (34.1%)	< 0.001

**Table 2:** Model performance metrics

Model	AUC (95% CI)	Sensitivity	Specificity	Precision (PPV)	F1-score	Accuracy
XGBoost-DART	0.881 (0.854–0.908)	0.831	0.809	0.542	0.656	0.814
Gaussian NB	0.872 (0.843–0.901)	0.856	0.762	0.494	0.626	0.782
LightGBM-DART	0.867 (0.838–0.896)	0.808	0.801	0.530	0.640	0.802
Random Forest	0.859 (0.829–0.889)	0.785	0.815	0.535	0.636	0.809
AdaBoost	0.843 (0.811–0.875)	0.771	0.798	0.513	0.616	0.792
MLP	0.834 (0.801–0.867)	0.748	0.806	0.519	0.613	0.794
SVM-RBF	0.825 (0.791–0.859)	0.734	0.811	0.521	0.609	0.795
KNN	0.812 (0.776–0.848)	0.717	0.793	0.489	0.581	0.777
Ridge	0.794 (0.756–0.832)	0.691	0.802	0.487	0.571	0.778

**Table 3:** Pairwise DeLong test comparing XGBoost-DART against other models

Comparison	ΔAUC	95% CI of ΔAUC	*P* value	*P* (Bonferroni)
XGBoost-DART vs. Gaussian NB	0.009	(− 0.018, 0.036)	0.513	1
XGBoost-DART vs. LightGBM-DART	0.014	(− 0.011, 0.039)	0.272	1
XGBoost-DART vs. Random Forest	0.022	(0.001, 0.043)	0.041	0.328
XGBoost-DART vs. AdaBoost	0.038	(0.012, 0.064)	0.004	0.032
XGBoost-DART vs. MLP	0.047	(0.019, 0.075)	0.001	0.008
XGBoost-DART vs. SVM-RBF	0.056	(0.026, 0.086)	< 0.001	0.003
XGBoost-DART vs. KNN	0.069	(0.037, 0.101)	< 0.001	< 0.001
XGBoost-DART vs. Ridge	0.087	(0.054, 0.120)	< 0.001	< 0.001

## Supplementary Information

Below is the link to the electronic supplementary material.

Supplementary Material 1

## Data Availability

Data used in this study were extracted from the publicly available Medical Information Mart for Intensive Care III (MIMIC-III) database. The database access details are available at https://physionet.org/content/mimiciii/1.4/. All code used for data analysis and model construction is available from the corresponding author on reasonable request.
