# Supplementary figures and images for: Early prediction of sepsis in the ICU: a comparative analysis of multiple machine-learning algorithms using the MIMIC-III database
Source: BMC Med Inform Decis Mak. 2026 Jun 5;26:275. doi: 10.1186/s12911-026-03610-1 (PMC13386605; doi:10.1186/s12911-026-03610-1)

# ROC Curves

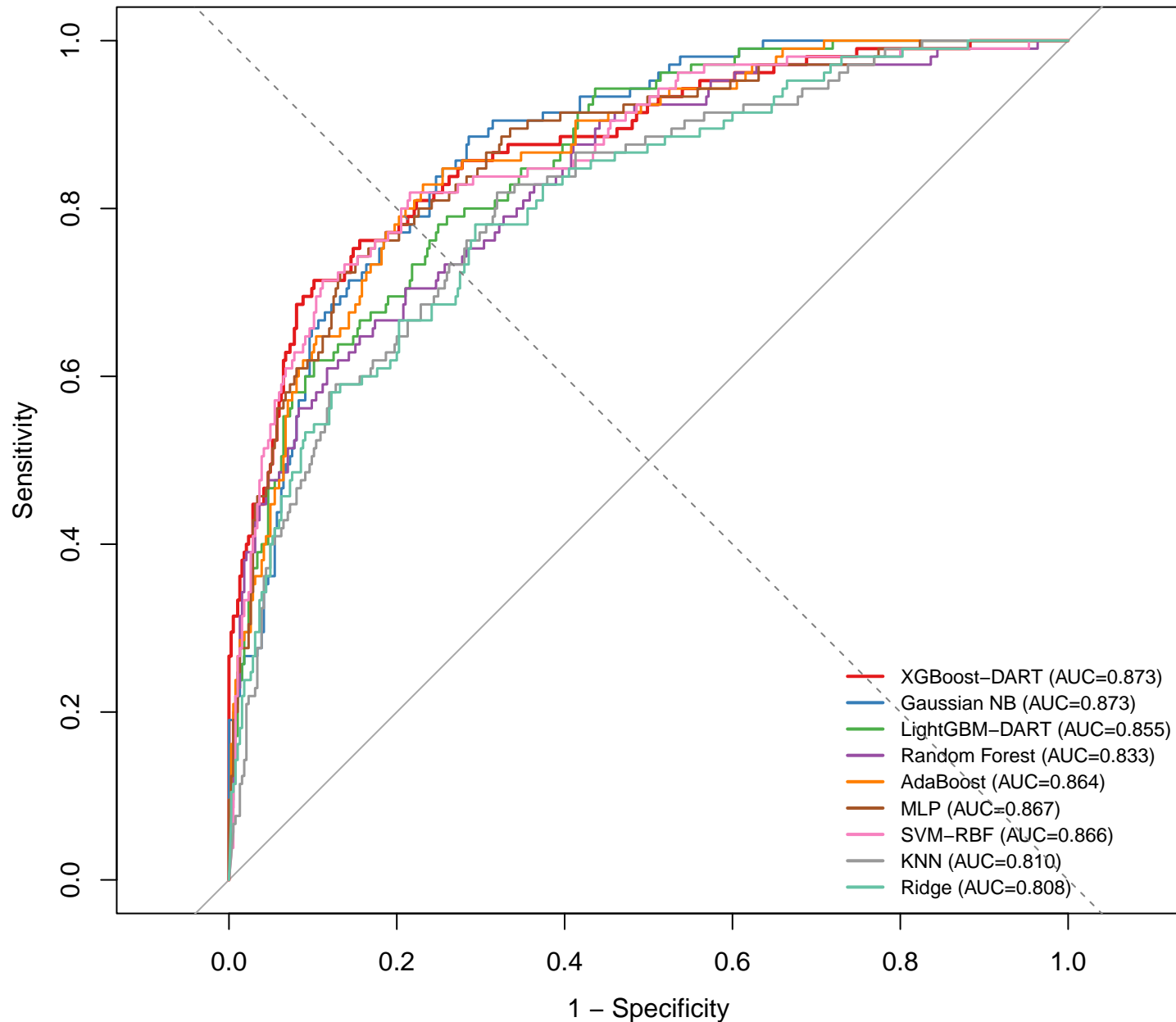

Supplement: Supplementary file 1 — Supplementary Material 1 [file 12911_2026_3610_MOESM1_ESM.zip › figures and tables/Figure4_ROC_curves.pdf]

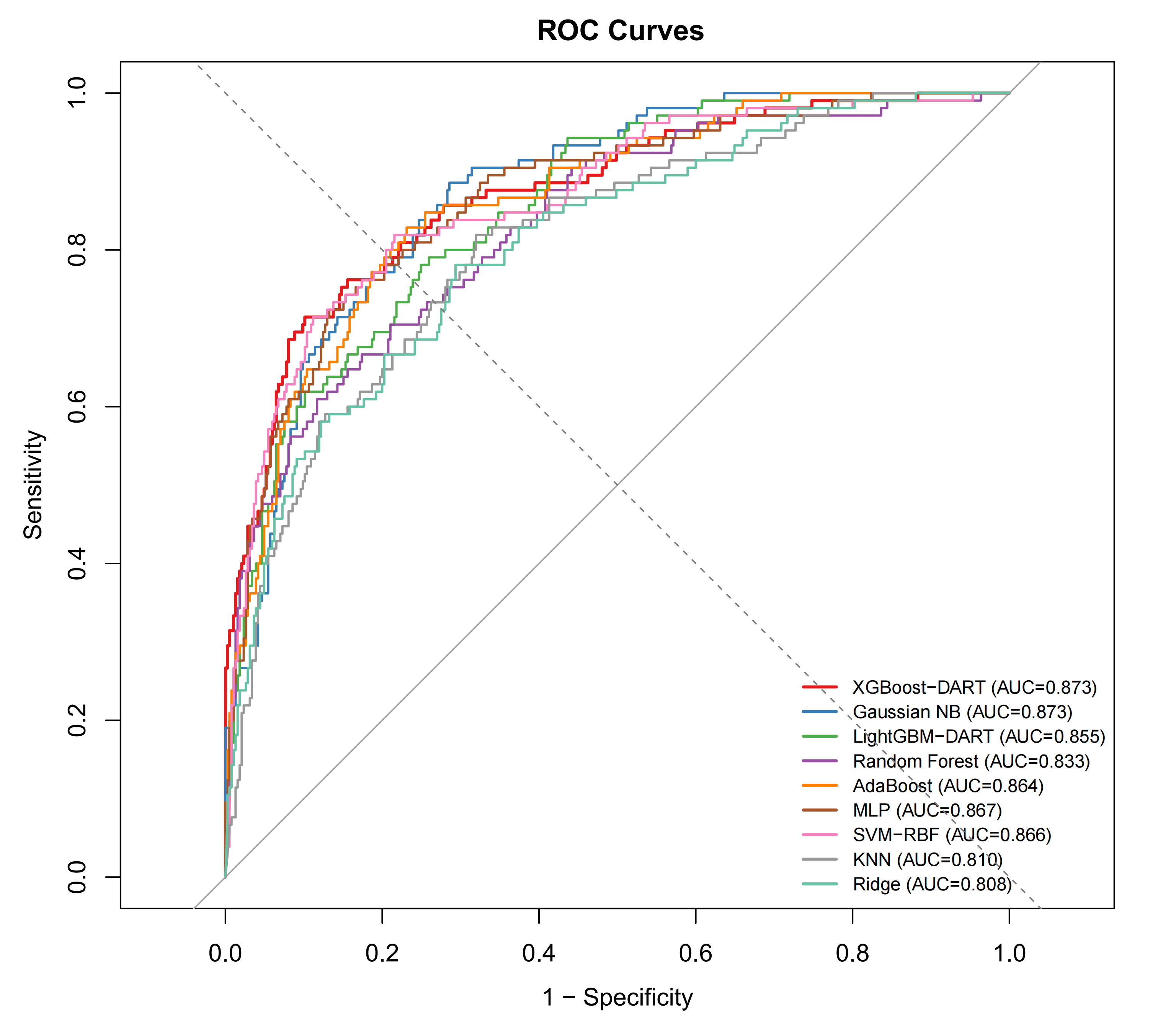

Supplement: Supplementary file 1 — Supplementary Material 1 [file 12911_2026_3610_MOESM1_ESM.zip › figures and tables/Figure4_ROC_curves.png]

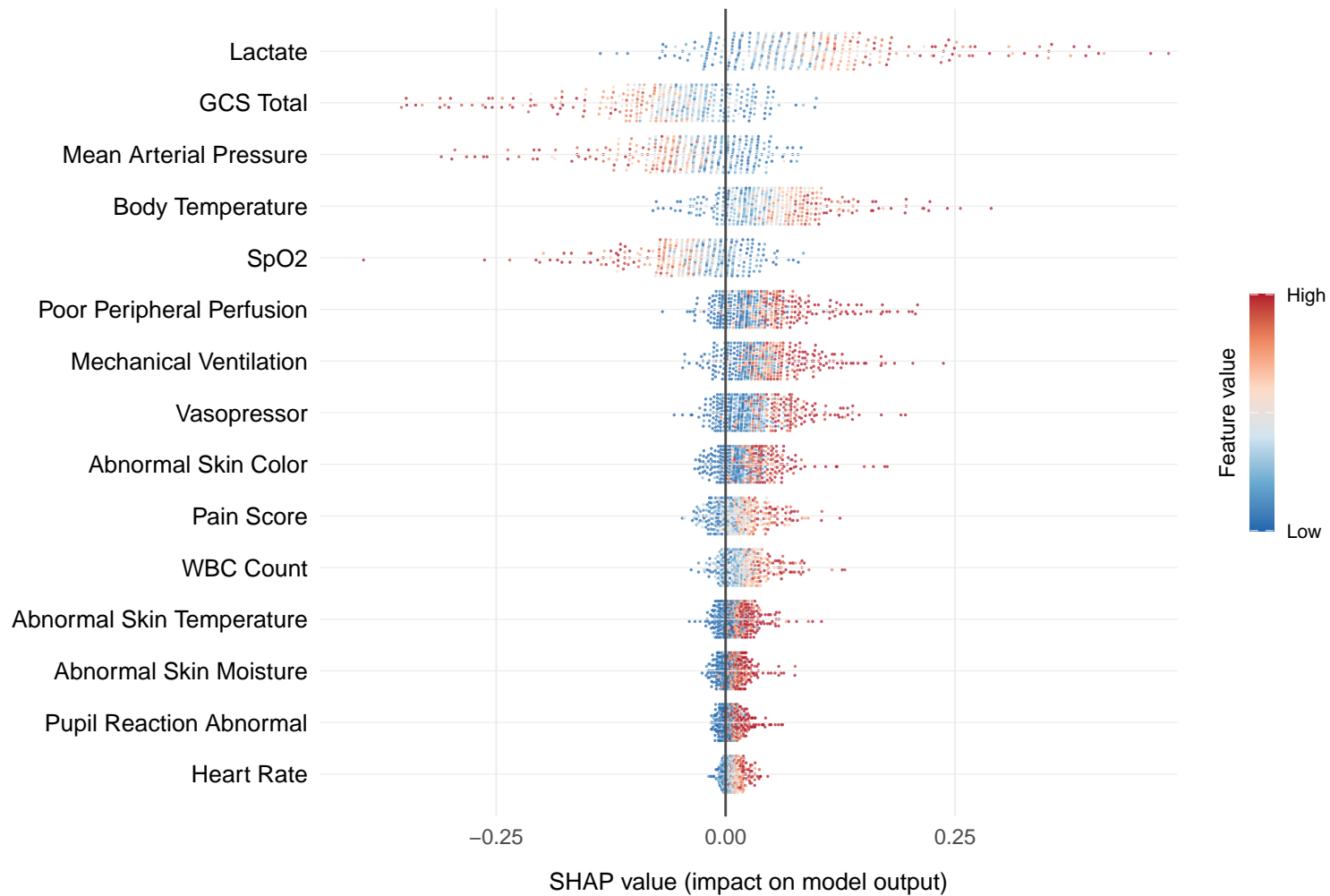

Supplement: Supplementary file 1 — Supplementary Material 1 [file 12911_2026_3610_MOESM1_ESM.zip › figures and tables/Figure5_SHAP_beeswarm.pdf]

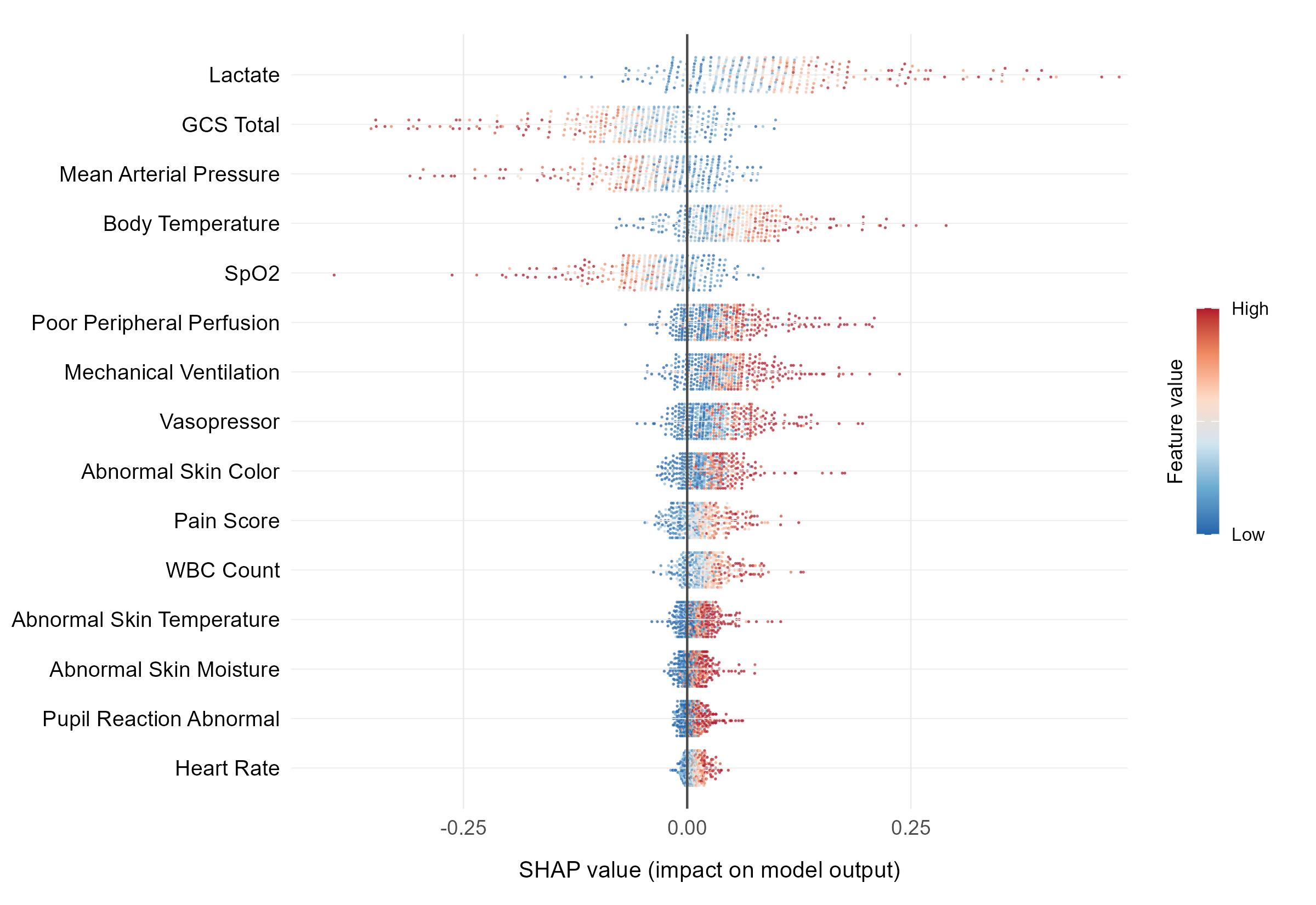

Supplement: Supplementary file 1 — Supplementary Material 1 [file 12911_2026_3610_MOESM1_ESM.zip › figures and tables/Figure5_SHAP_beeswarm.png]

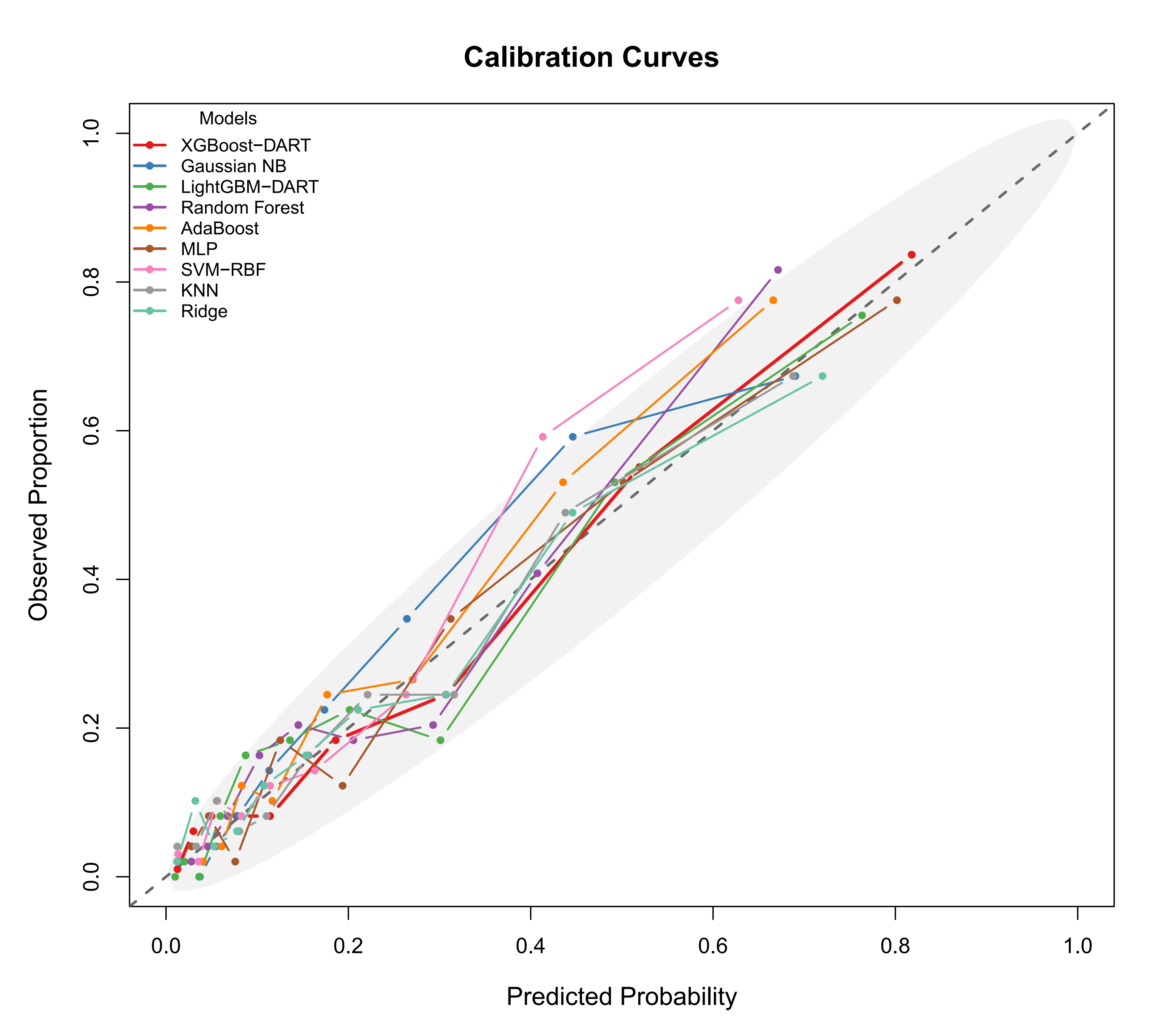

Supplement: Supplementary file 1 — Supplementary Material 1 [file 12911_2026_3610_MOESM1_ESM.zip › figures and tables/Figure6_Calibration_curves.png]

# Calibration Curves

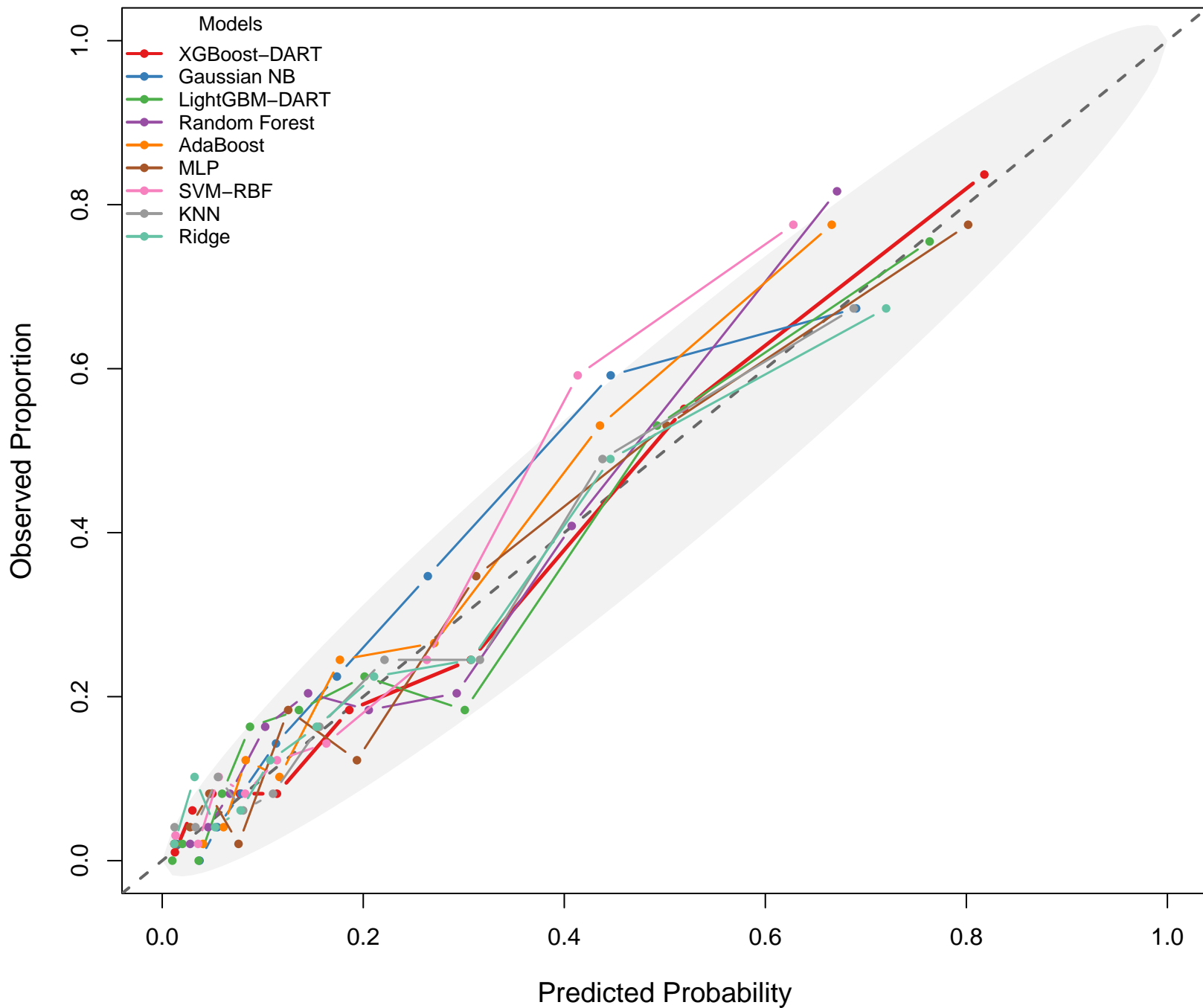

Supplement: Supplementary file 1 — Supplementary Material 1 [file 12911_2026_3610_MOESM1_ESM.zip › figures and tables/Figure6_Calibration_curves_.pdf]
